# Supplementary material for: A descriptive marker gene approach to single-cell pseudotime inference
Source: Bioinformatics. 2018 Jun 23;35(1):28–35. doi: 10.1093/bioinformatics/bty498 (PMC6298060; doi:10.1093/bioinformatics/bty498)
Supplement: Supplementary Data [file bty498_suppl_data.pdf]

# Supplementary Information

Kieran Campbell
University of Oxford

Christopher Yau
University of Birmingham

February 5, 2018

## Contents

**1 Supplementary Figures** 3

**2 Supplementary Tables** 12

**3 Data preprocessing** 13

**4 Modelling considerations** 13
4.1 Mean-variance relationship . . . . . . . . . . . . . . . . . . . . . . . . . 13
4.2 Dropout rate . . . . . . . . . . . . . . . . . . . . . . . . . . . . . . . . 14

**5 Further model description** 16

**6 Leave-$k$-out validation of marker pseudotime fits** 17

**7 Marker-gene pseudotime inferene does not preclude whole-transcriptome analysis** 17

**8 Description of whole-transcriptome algorithms** 21

**9 Simulating data** 22

**10 Incorporating prior information can improve pseudotime inference** 22

**11 Benchmarking** 24
11.1 Mean functions . . . . . . . . . . . . . . . . . . . . . . . . . . . . . . . 24
11.1.1 Sigmoidal . . . . . . . . . . . . . . . . . . . . . . . . . . . . . . 24
11.1.2 Complementary log-log . . . . . . . . . . . . . . . . . . . . . . . 25
11.1.3 Probit . . . . . . . . . . . . . . . . . . . . . . . . . . . . . . . . 25
11.1.4 Threshold . . . . . . . . . . . . . . . . . . . . . . . . . . . . . . 25
11.2 Pseudotime inference . . . . . . . . . . . . . . . . . . . . . . . . . . . . 26

**12 Markers used** **26**

The entire analysis is available as a reproducible workflow at `http://www.github.com/kieranrcampbell/ouija-paper` but we summarise some steps here.

# 1 Supplementary Figures

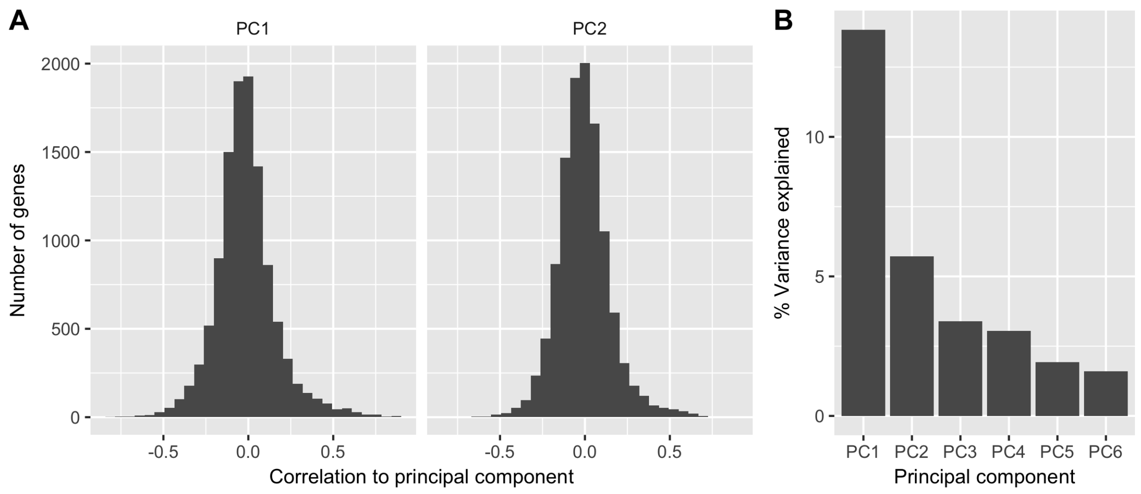


**Supplementary Figure 1:** Compressibility of Trapnell et al. (2014) dataset. **A** The expression patterns of many genes are highly correlated with the first and second principal components of the data. **B** Variance explained by each PC.

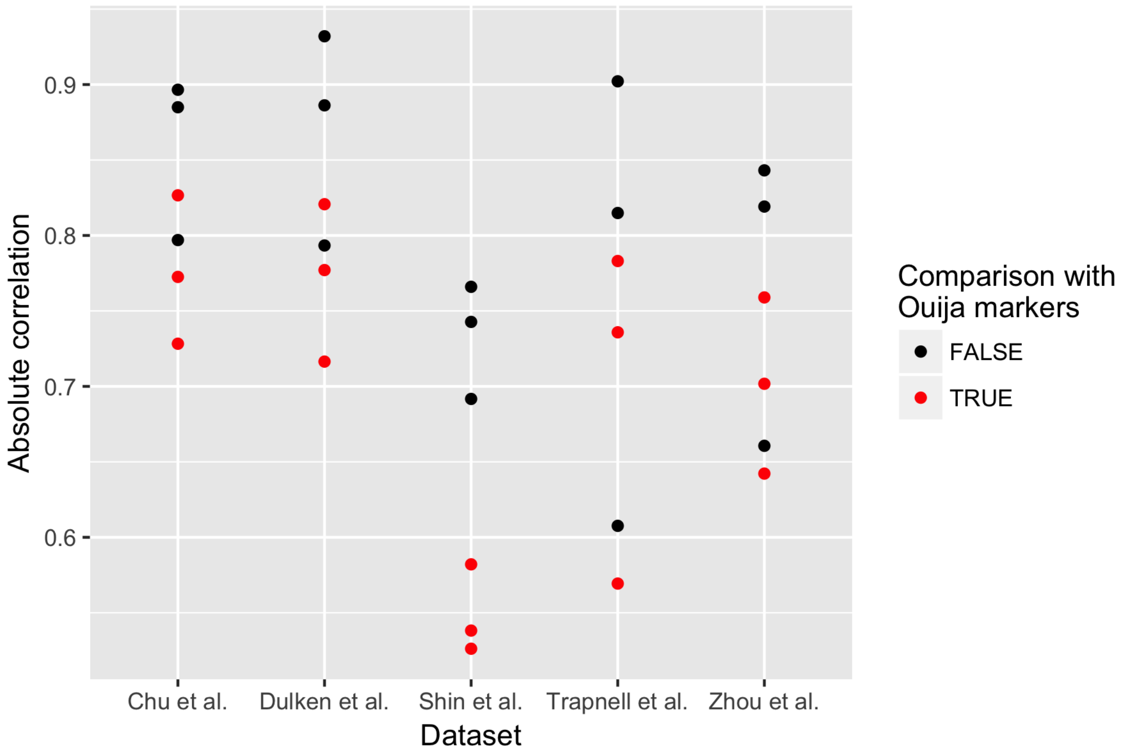


**Supplementary Figure 2:** Comparison of DPT, TSCAN and Monocle 2 whole transcriptome derived pseudotimes. Black shows correlations between DPT-TSCAN, DPT-Monocle 2 and TSCAN-Monocle 2. Red show correlations of each algorithm with marker-based Ouija estimates.

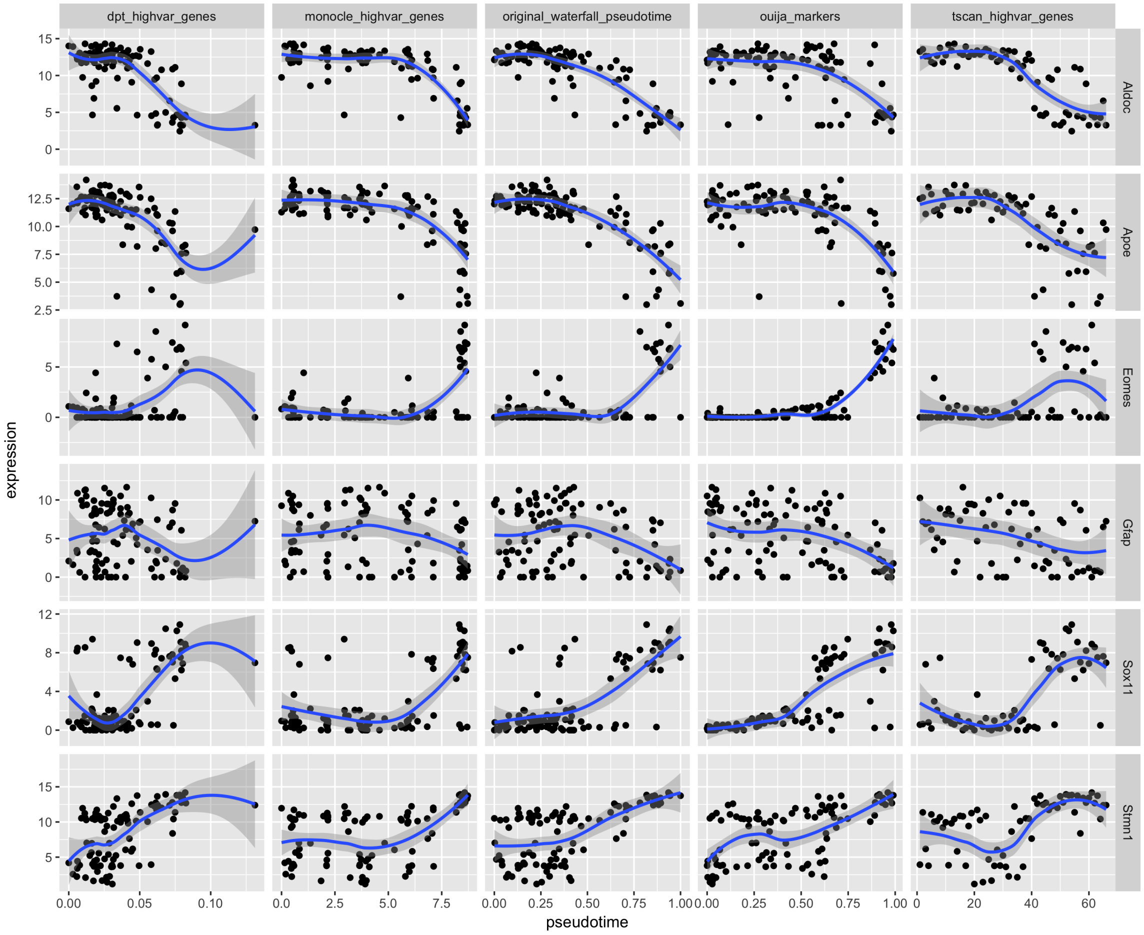


**Supplementary Figure 3:** Inferred pseudotemporal behaviour of marker genes from Shin *et. al.* (2015).

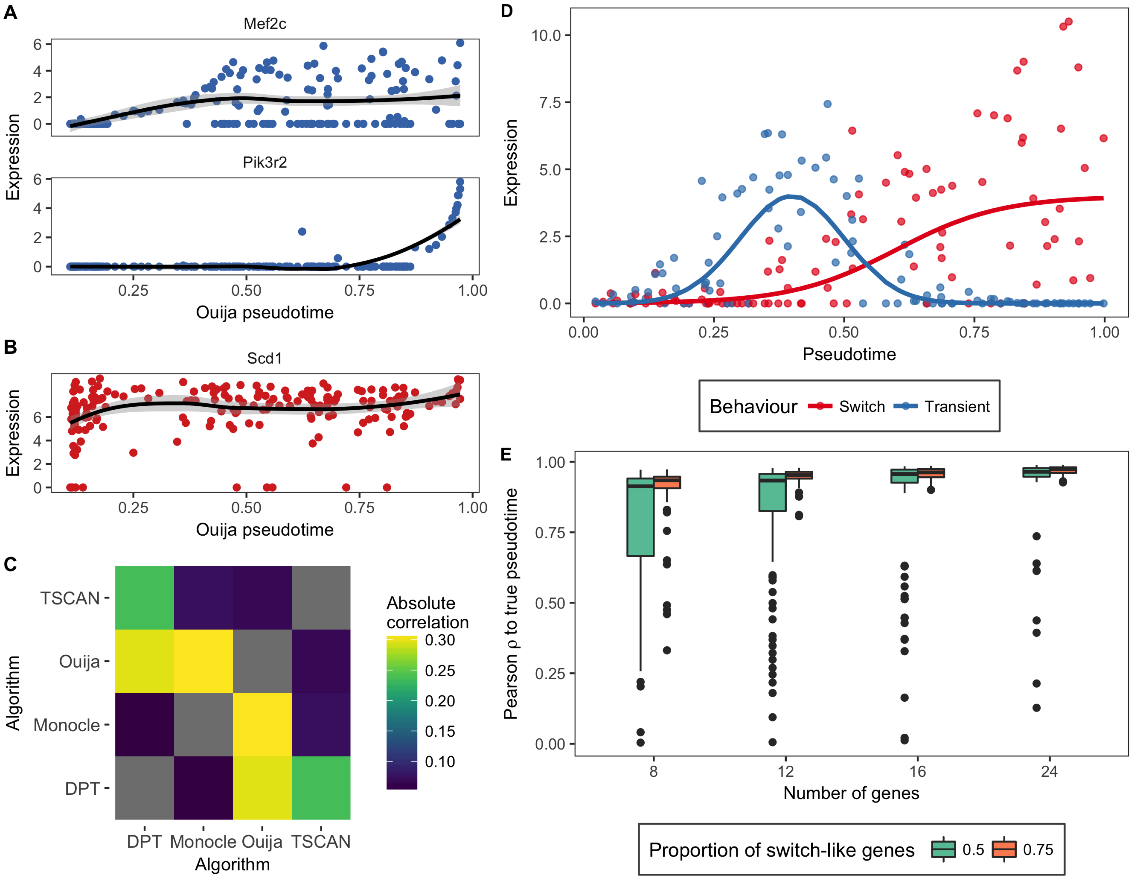


**Supplementary Figure 4: Ouija is robust to gene behaviour misspecification.** **A** The genes *Mef2c* and *Pik3r2* show the expected behaviour in a marker-based pseudotime fitted to the Li *et al.* (2016) [1] dataset ("constant upregulation" and "transient upregulation" respectively). However, the gene *Scd1* (**B**) was claimed to have "tide wave" regulation (transient expression), but a LOESS fit over pseudotime (black line) shows effectively constant expression over pseudotime. **C** We found very low agreement between the different pseudotime inference algorithms for this dataset. Curiously, the largest agreement was reported between Ouija using only markers and Monocle 2 using the 500 most variable genes. **D** We simulated datasets with genes either exhibiting switch-like expression over pseudotime or transient expression, with an overdispersed, zero-inflated noise model to mimic real data. **E** Ouija was benchmarked assuming all genes were switch-like when a certain proportion were actually transient across a range of geneset sizes. Even at only 8 genes, half of which are actually transient, Ouija still recovers a median correlation of greater than 0.9 with the true pseudotime, which only increases with increasing number of genes and switch-like behaviour.

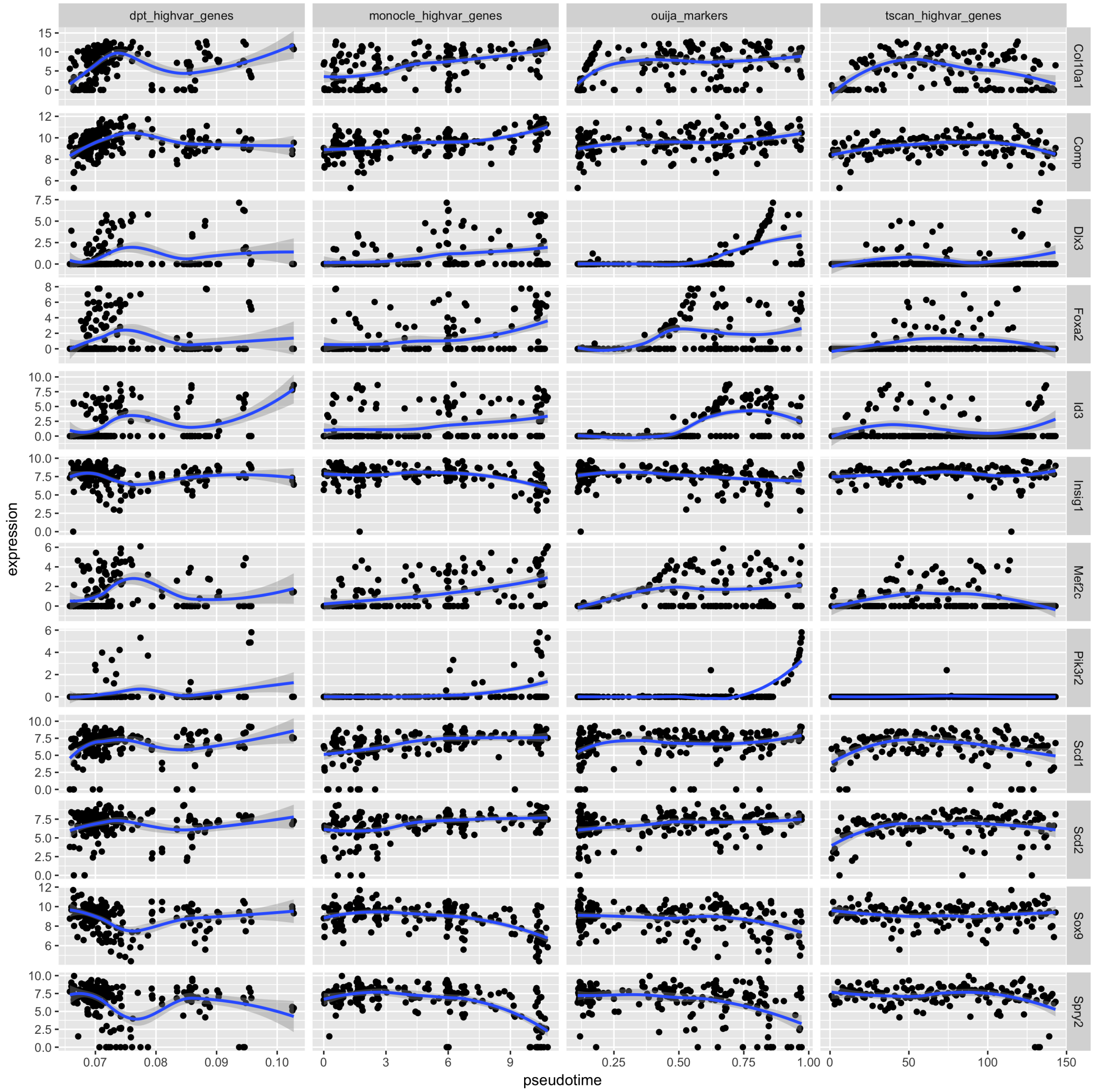


**Supplementary Figure 5:** Inferred pseudotemporal behaviour of marker genes from Li *et. al.* (2016).

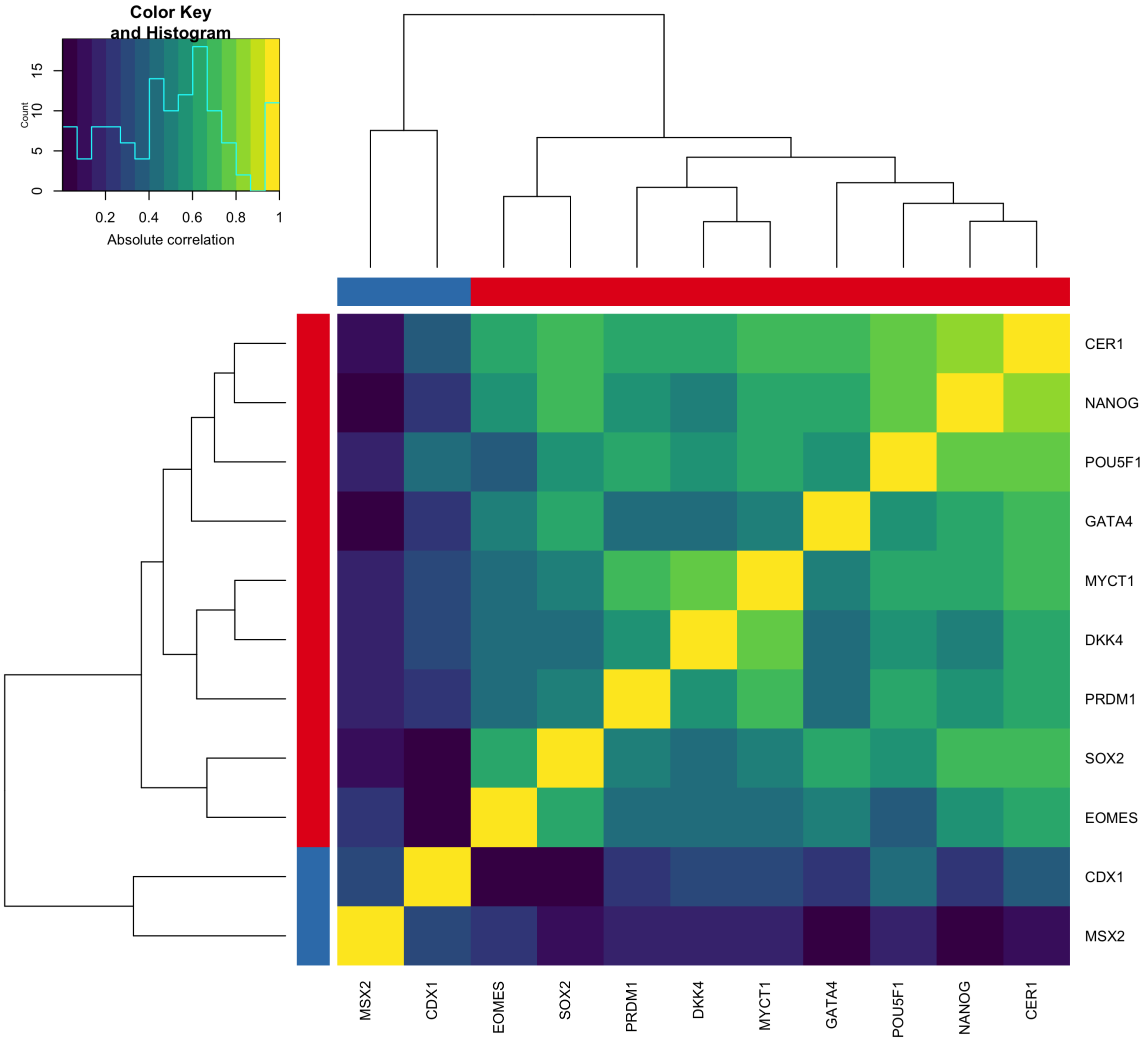


**Supplementary Figure 6:** Absolute correlation of marker genes in the Chu *et. al.* (2016) dataset reveal clustering into transient genes (blue) and switch-like genes (red).

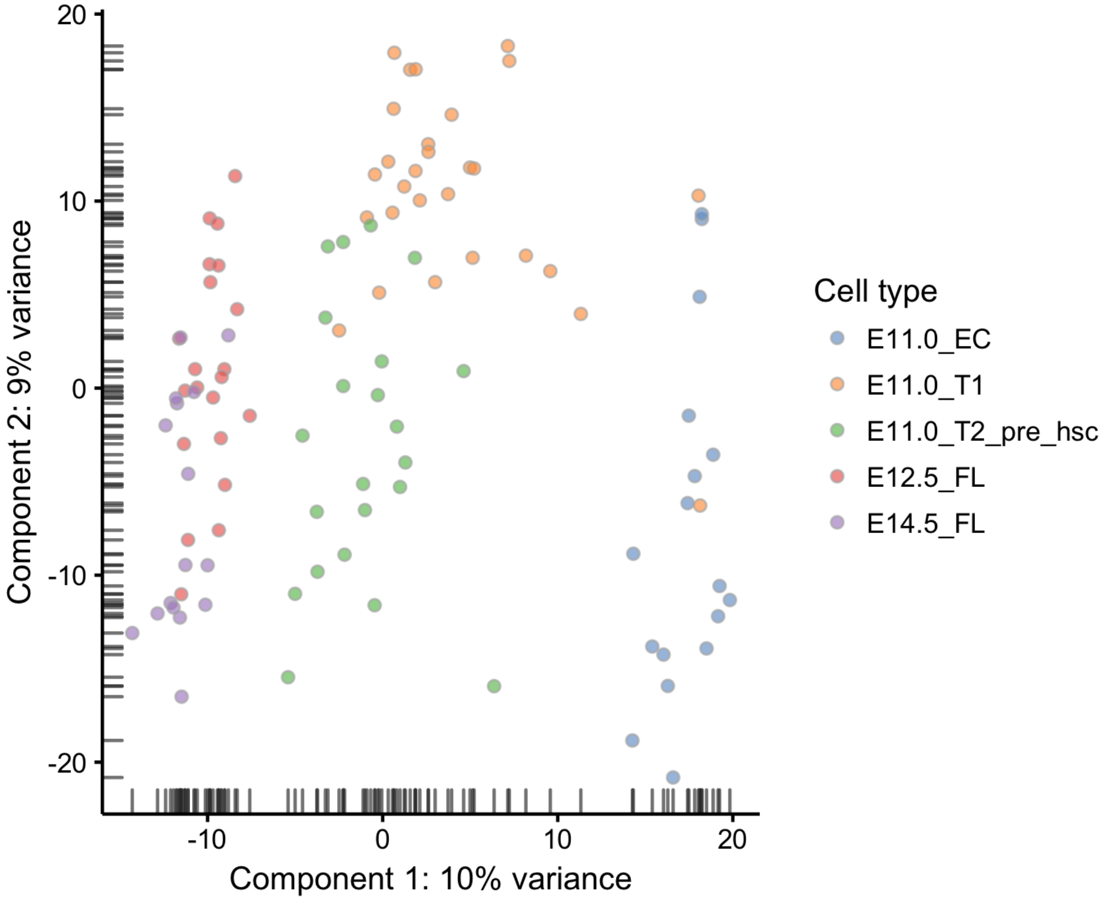


**Supplementary Figure 7:** PCA representation of Zhou *et. al.* (2016) dataset using 1,000 most variable genes, coloured by cell type.

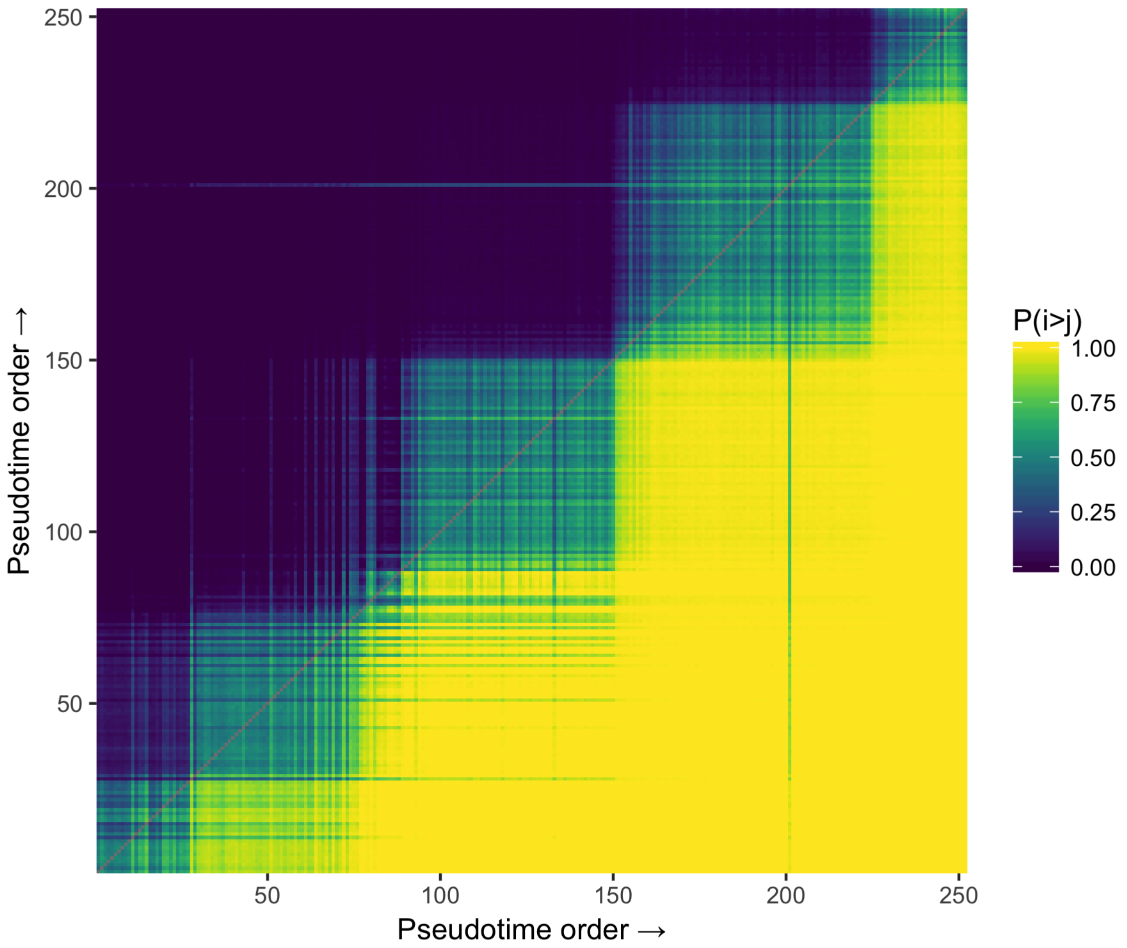


**Supplementary Figure 8:** Consistency matrix for clustering with the Dulken *et. al.* (2017) dataset.

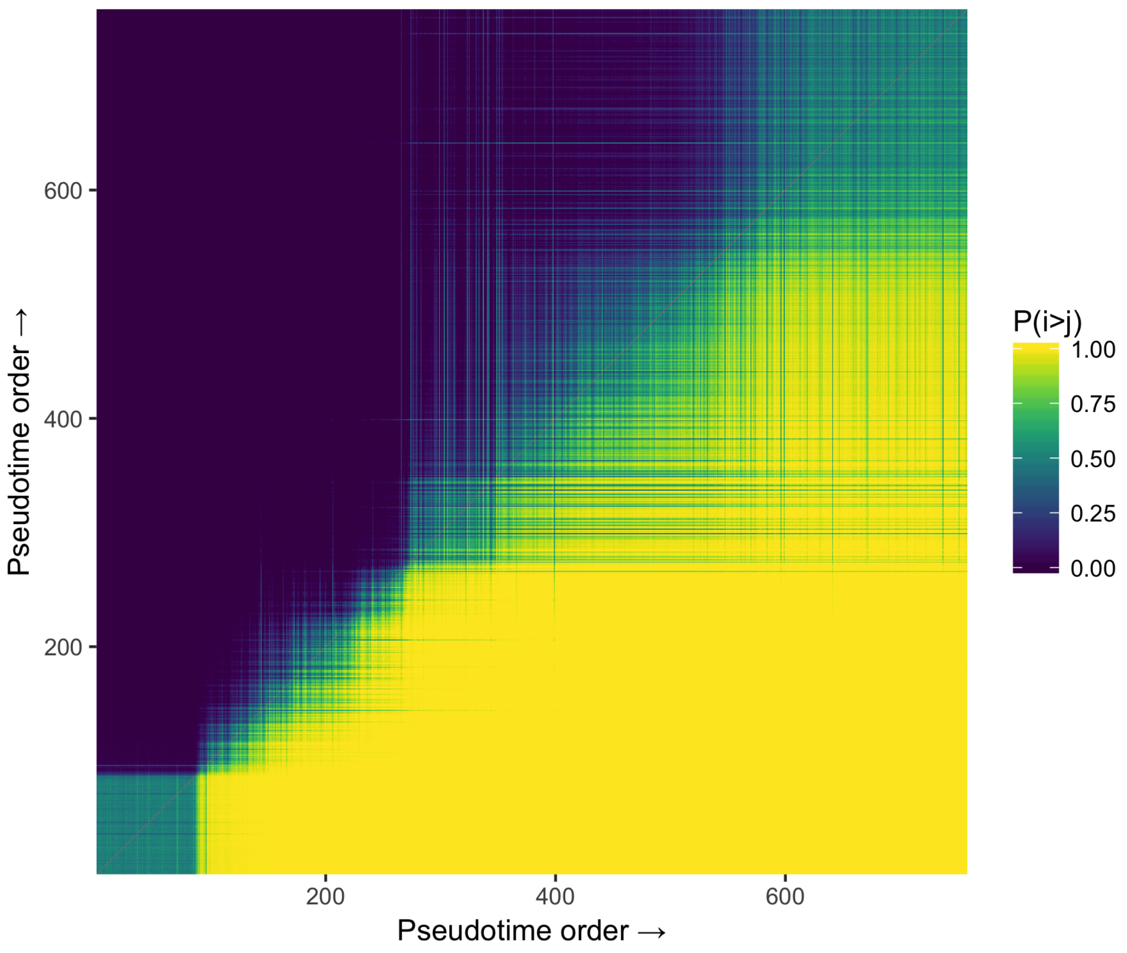


**Supplementary Figure 9:** Consistency matrix for clustering with the Chu *et. al.* (2016) dataset.

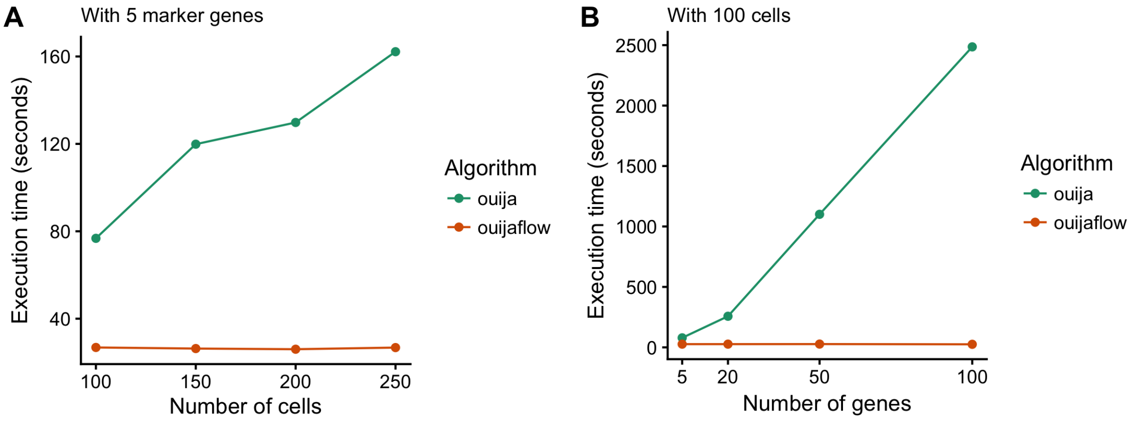


**Supplementary Figure 10:** Example run times on a 2014 MacBook Pro of Ouija and Ouijaflow for the cell cycle dataset for differing numbers of cells (**A**) and genes (**B**), for 3000 iterations of Ouija and 1000 iterations of Ouijaflow. Both algorithms require variable numbers of iterations to be considered converged; users should inspect MCMC diagnostics such as effective sample size for Ouija; users should check that the lower bound has approximately converged for Ouijaflow. Note that HMC (used in Ouija) has a data-dependent clock time per iteration.

# 2 Supplementary Tables

| | 1 | 2 | 3 | 4 | 5 | 6 | 7 |
|---|---|---|---|---|---|---|---|
| aNSC | 3 | 0 | 21 | 55 | 15 | 46 | 12 |
| NPC | 2 | 0 | 2 | 2 | 1 | 11 | 13 |
| qNSC | 22 | 33 | 10 | 0 | 1 | 1 | 2 |

**Supplementary Table 1:** Comparison of experimentally measured cell types (rows) and Ouija-inferred cell types (columns) for the Dulken et al. dataset.

| | 1 | 2 | 3 | 4 | 5 | 6 | 7 | 8 |
|---|---|---|---|---|---|---|---|---|
| 00h | 81 | 11 | 0 | 0 | 0 | 0 | 0 | 0 |
| 12h | 0 | 42 | 60 | 0 | 0 | 0 | 0 | 0 |
| 24h | 0 | 0 | 65 | 0 | 1 | 0 | 0 | 0 |
| 36h | 0 | 0 | 17 | 47 | 70 | 25 | 10 | 3 |
| 72h | 0 | 0 | 0 | 0 | 33 | 42 | 18 | 45 |
| 96h | 0 | 0 | 0 | 0 | 45 | 38 | 36 | 69 |

**Supplementary Table 2:** Comparison of cell capture times (rows) and Ouija-inferred cell types (columns) for the Chu et al. dataset.

| Number of genes | Simulation regime | $p$-value |
|---|---|---|
| 6 | Sigmoidal | 2.6E-03 |
| 6 | Complementary log-log | 1.3E-05 |
| 6 | Probit | 1.3E-04 |
| 6 | Threshold | 5.9E-28 |
| 9 | Sigmoidal | 1.8E-04 |
| 9 | Complementary log-log | 6.7E-07 |
| 9 | Probit | 3.3E-09 |
| 9 | Threshold | 3.3E-12 |
| 12 | Sigmoidal | 2.0E-03 |
| 12 | Complementary log-log | 1.8E-06 |
| 12 | Probit | 2.5E-08 |
| 12 | Threshold | 9.2E-06 |
| 15 | Sigmoidal | 3.0E-03 |
| 15 | Complementary log-log | 4.2E-06 |
| 15 | Probit | 2.0E-09 |
| 15 | Threshold | 6.7E-03 |

**Supplementary Table 3:** P-values reported by Wilcoxon rank-sum test on the correlations to true pseudotime comparing informative priors to noninformative priors.

# 3 Data preprocessing

**Trapnell et al.** Data were downloaded from the Bioconductor package HSMMSingleCell at http://bioconductor.org/packages/devel/data/experiment/html/HSMMSingleCell.html. Cells were filtered to contain any with State of 1 or 2. $\log_2$ FPKM $+ 1$ values were used as expression. Genes were filtered to include any with a variance in expression greater than 1.

**Shin et al.** TPM expression values were downloaded from http://www.cell.com/cms/attachment/2038326541/2052521610/mmc7.xlsx. $\log_2$ TPM $+ 1$ values were used as expression. Genes were filtered to include any with a variance in expression greater than 1.

**Zhou et al.** FPKM values were downloaded from the Gene Expression Omnibus with accession GSE67120. Cells were filtered to include only those with the desired cell type mentioned in the main text. $\log_2$ FPKM $+ 1$ values were used as expression.

**Dulken et al.** TMM-normalised counts were downloaded from http://www.cell.com/cms/attachment/2081925929/2072608613/mmc5.xlsx. Expression values used were $\log_2(\text{Norm-counts} + 1)$. Cells were filtered to be of types qNSC, aNSC or NPC.

**Chu et al.** Raw counts were downloaded from the Gene Expression Omnibus with accession GSE75748. Counts were normalised using TMM normalisation. Expression values used were $\log_2(\text{Norm-counts} + 1)$

**Li et al.** Log expression values were downloaded from the Gene Expression Omnibus with accession GSE76157. Outlier cells were removed in a similar manner to the original publication.

# 4 Modelling considerations

## 4.1 Mean-variance relationship

In normalised RNA-seq counts the variance for gene $g$ in sample $n$ is related to its mean via

$$\sigma^2_{ng} = \mu_{ng}(1 + \phi_g \mu_{ng}) \tag{1}$$

where $\phi_g$ is a gene-specific dispersion factor. Such strong parametric forms are typically required since for low sample sizes the estimates of the variance can be very unstable.

However, typically in single-cell data there are enough measurements (ie cells) to allow robust estimation of both the mean and variance for each gene [2]. The exception to this is in pseudotime analyses, where we are assuming each cell represents a unique time point, and therefore the mean $\mu_{ng}$ and variance $\sigma^2_{ng}$ are effectively measured only once. Consequently we must consider a strong mean-variance relationship since assuming a constant variance per gene is akin to under-fitting while it would be impossible to fit a variance for each cell and each gene (since there is only one measurement).

As a solution to this we examine the mean-variance relationship for the genes across all cells and assume the same relationship approximately holds for cells as they progress along pseudotime trajectories. The relationship in 1 applies to the original untransformed data (e.g. TPM or scaled counts) while we wish to model the $\log_2(\text{TPM} + 1)$ transformed relationship directly. Therefore we must examine the mean-variance relationship for the $\log_2$ data[1], since in general the mean-variance relationship of the log-transormed data isn't the same as (the log of) the mean-variance relationship on the untransformed data.

We examined the mean-variance relationship of the logged data for the three datasets in the main text, as seen in Supplementary Text Figure 1. The pseudotemporal marker genes identified in the original text are shown in red. For both the Trapnell and Zhou datasets these lie on the 'leading edge' of the relationship, in areas of moderate mean expression but high variance. In comparison, the housekeeping genes (shown in blue) lie in the tails in regions of high mean expression but low overall variance. This makes intuitive sense, as we expect the marker genes to turn on across the trajectory, giving them a mean of around half the maximal value but maximum variance. In contrast, we expect housekeeping genes to have maximal expression across the trajectory but very low variance in keeping with the constancy of their expression. Oddly we found such a relationship not to hold in the Shin et al. dataset.

We therefore assumed that any genes we wish to model as pseudotemporal marker genes follow the same linear mean-variance relationship. To quantify this for generating synthetic data we fit a simple linear model with a forced zero intercept to the pseudotemporal marker genes in the Trapnell et al. dataset that gave a gradient of $3.502$. Therefore, in all our synthetic data we model $\sigma^2 = 3.5\mu$.

## 4.2 Dropout rate

It has been noted in several papers (see e.g. [3]) that the dropout rate for a given gene is inversely proportional to the mean expression of that gene. This is typically assumed to be due to the failure of reverse transcription of lowly expressed transcripts. To account for this we assume that the probability of a dropout is logistic on the latent gene expression mean in a similar approach to Kharchenko et al 2014. The difference to previous approaches is

[1] Note for $x \gg 0$ $\log_2(x + 1) \approx \log_2(x)$.

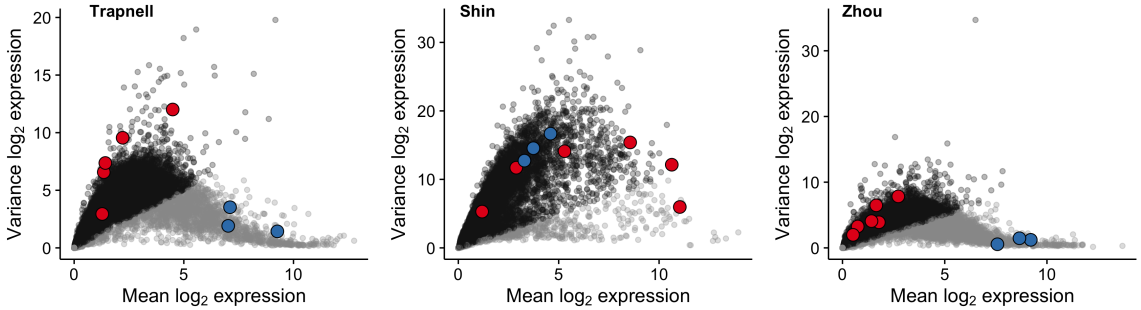


**Supplementary Text Figure 1:** The mean-variance relationship in $\log_2(\mathrm{TPM}+1)$ single-cell data for three of the datasets studied in the main text. Red denotes the marker genes identified in the main text while blue corresponds to three house-keeping genes (*LDHA*, *NONO*, *PGK1*) or their mouse equivalents. Both the Trapnell *et. al.* (2014) and Zhou *et. al.* (2016) datasets show consistent evidence that pseudotemporal marker genes exist on the 'leading edge' of the data with medium mean expression but high variance. This suggests a linear relationship between mean and variance in $\log_2$ space. In contrast, the housekeeping genes all sit in the 'tails' with high mean expression but very low variance.

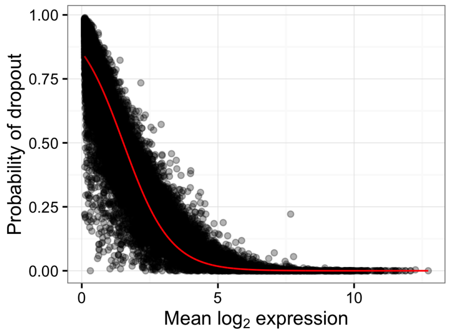


**Supplementary Text Figure 2:** The probability of a dropout against the mean $\log_2$ expression in the Trapnell *et. al.* (2014) dataset. The red solid line shows the logistic regression fit.

that (1) we are working in $\log_2(\text{TPM} + 1)$ space and (2) we assume a unique mean $\mu_{ng}$ for every gene in every cell, giving a per-gene per-cell dropout probability $p(\mu_{ng})$ of

$$\log\left[\frac{p(\mu_{ng})}{1 - p(\mu_{ng})}\right] = \beta_0 + \beta_1 \mu_{ng}. \tag{2}$$

During statistical inference $\beta_0$ and $\beta_1$ are assumed to be constant across all cells as the (assumed) small number of marker genes used would make per-gene inference unstable. To generate synthetic data we fit a logistic regression curve to the probability of dropout against mean $\log_2$ expression for the Trapnell et al dataset (Supplementary Text Figure 2). This gave coefficients of $\beta_0 = 1.763$ and $\beta_1 = -1.156$.

# 5 Further model description

Suppose we model the gene expression $y$ of some dynamically unfolding process at time $t$. $y$ is fundamentally a random variable so we model its expected expression $\mathbb{E}[y(t)] = f(t)$, where $f$ is a function that dictates the mean gene behaviour over time. The difficulty with pseudotime analysis is that $t$ is unknown and the object of inference, so if we are to infer $t$ we must make assumptions about $f$ for $t$ to be identifiable.

The question of pseudotime modelling then comes down to deciding a form of $f$. One possibility is a linear function $f(t) = at + b$ for parameters $a$ and $b$. However, this is fundamentally unrealistic - it can model negative expression, which doesn't make physical sense, and also unbounded expression, i.e. as the dynamical process time $t$ increases the expression goes to infinity. On the other hand we could not fix a functional form of $f$ and model it nonparametrically, such as using a Gaussian process (GP, see e.g. [4]). However, using a GP does not lead to any interpretable understanding of gene behaviour without post-hoc processing and is typically nonidentifiable, requiring capture time priors such as in [5].

In Ouija, we choose a form of $f$ that models realistic gene behaviours over time allowing for flexible modelling of transcription dynamics without the overflexibility of nonparametric functions. Specifically, $f$ can be sigmoidally regulated with parameters $k$ and $t^{(0)}$ corresponding to switch strengths and switch times, or transiently regulated with parameters $p$ and $b$ corresponding to the peak times and length of upregulation respectively.

Suppose we have $n \in 1, \ldots, N$ cells and $g \in 1, \ldots, G$ genes for which we have an expression measurement $y_{ng}$ and for each cell we wish to associate a pseudotime $t_n$. For each gene we have a set of gene specific parameters $\Theta_g$, which will either be switch times/strengths or peak times/lengths depending on whether we model gene $g$ as switch or transient. Given a student-t distribution $\text{T}_\nu$ and a per-sample variance $\sigma^2_{ng}$ (as detailed in the main text) then the likelihood for a single observation is given by

$$p(y_{ng} | f(t_n, \Theta_g), \sigma^2_{ng}) = \text{T}_\nu(y_{ng} | f(t_n, \Theta_g), \sigma^2_{ng}). \tag{3}$$

The observations are conditionally independent given the gene parameters and pseudotimes, so the joint probability of the data $\boldsymbol{Y}$, gene specific parameters, and pseudotimes is given by

$$p(\boldsymbol{Y}, \boldsymbol{\Theta}, \boldsymbol{t}) = \pi(\boldsymbol{\Theta})\pi(\boldsymbol{t}) \prod_{g=1}^{G} \prod_{n=1}^{N} \mathrm{T}_{\nu}(y_{ng} | f(t_n, \Theta_g), \sigma^2_{ng}) \tag{4}$$

where $\pi(\boldsymbol{\Theta})$ and $\pi(\boldsymbol{t})$ are the prior distributions of the gene specific parameters and pseudotimes respectively. Using Bayes' rule this joint probability can be transformed into a posterior distribution of the pseudotimes and gene specific parameters given the data, but integration of the normalizing constant is intractable so we resort to approximate inference methods like MCMC and variational Bayes, as detailed in the main text. As such, this model is a form of Bayesian nonlinear factor analysis where the nonlinearities are induced by the functions $f$, the latent variables are the pseudotimes $t$, and the equivalent of the factor loadings are the interpretable gene-specific parameters.

# 6 Leave-$k$-out validation of marker pseudotime fits

Even though Ouija fits pseudotimes to small panels of marker genes whose *a priori* expectations of behaviour is typically taken to validate whole transcriptome pseudotime fits, it is still possible to use these genes to validate the pseudotimes using leave-$k$-out validation. We demonstrated this on the Trapnell et al. dataset by reinferring the pseudotimes (a) 5 times leaving 1 gene out and fitting the pseudotimes to the remaining 4 marker genes, and (b) 10 times leaving 2 genes out and fitting the pseudotimes to the remaining 3 genes. The behaviour of the held-out genes was then examined to ensure that it was consistent with the all-marker pseudotime fit and the a priori expectations of the researcher.

The results for the leave-one-out and leave-two-out analyses can be seen in Supplementary Text Figure 3 A & B respectively. In every case the behaviour of the held-out genes follows the *a priori* expectations exactly and matches with the behaviour inferred using the all-marker-fit. We interpret this as evidence that Ouija is robust to the exact selection of marker genes and that leave-$k$-out validation is an appropriate method to critique the pseudotime fits of Ouija, if desired.

# 7 Marker-gene pseudotime inferene does not preclude whole-transcriptome analysis

While Ouija fits pseudotimes to small sets of marker genes this in no way precludes whole-transcriptome analysis of the remaining genes. Ouija pseudotimes were fitted to the Trapnell et al. dataset, with 1500 post-burn-in MCMC iterations retained. 100 of iterations were randomly sampled giving an empirical estimate of the posterior pseudotime distribution

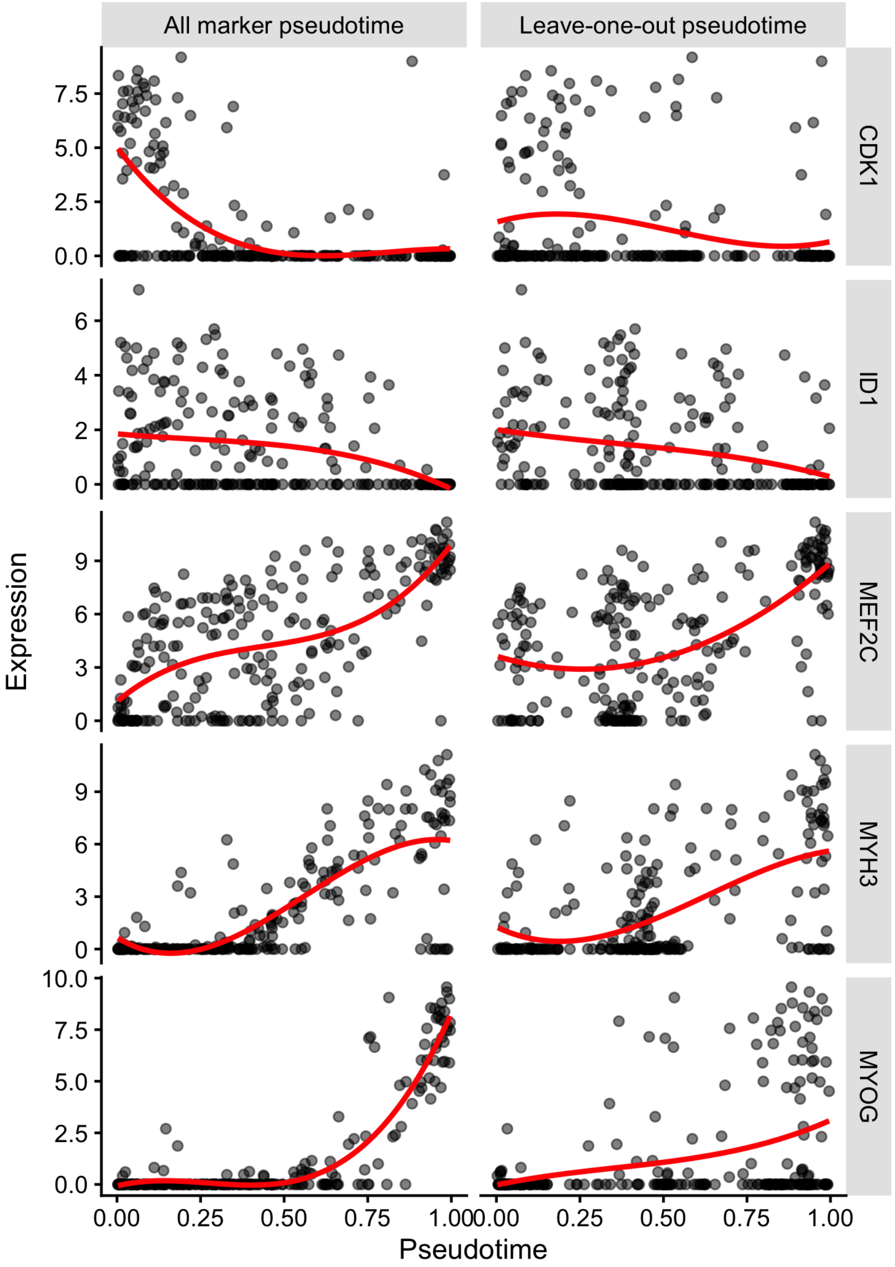


**Supplementary Text Figure 3:** .

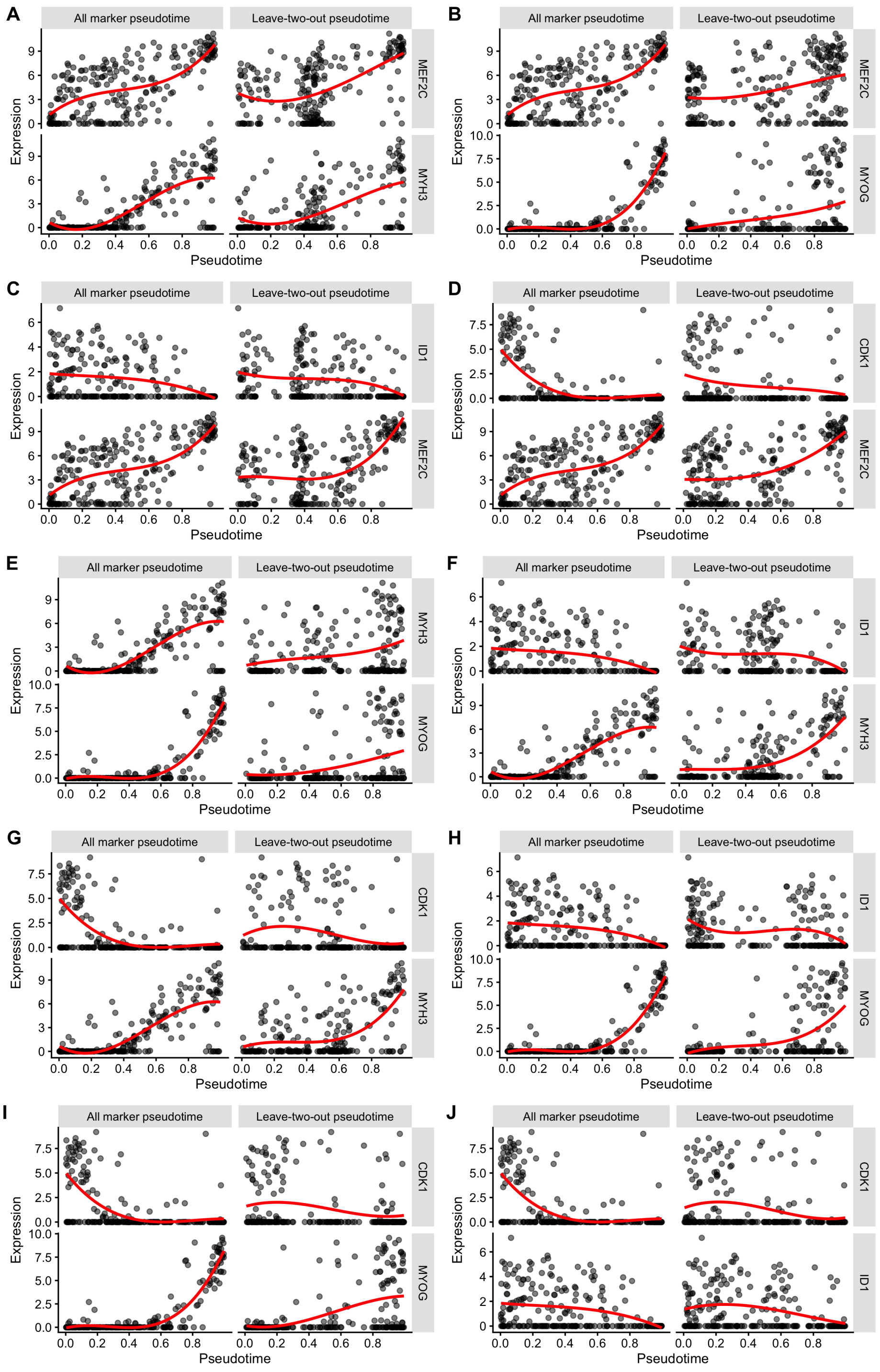


**Supplementary Text Figure 4:** .

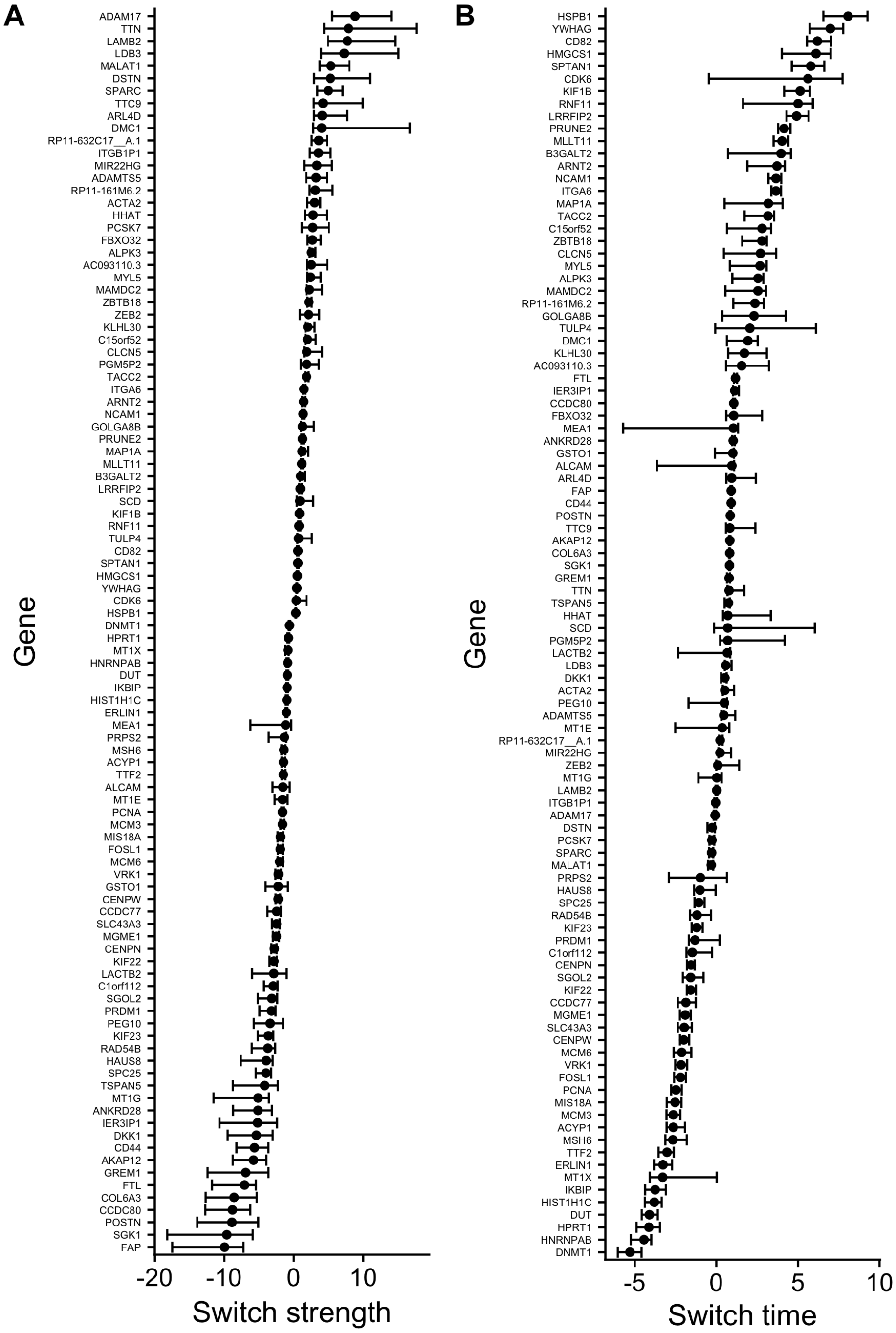


**Supplementary Text Figure 5:** For the Trapnell et al. dataset, sigmoidal regulation patterns were fitted to the transcriptome-wide gene-set after marker-gene pseudotime inference. Genes displayed are those significant across all posterior pseudotime traces, ordered by switch strength (**A**) and by switch time (**B**).

$p(t|\text{marker genes})$. For each of these 100 iterations we fitted `switchde` differential expression models to 11,257 genes passing the filtering threshold. This provided an estimate of the distribution of the switch strength ($k$) and time ($t^{(0)}$) parameters for all remaining genes in the transcriptome.

For illustrative purposes, we filtered down to the 875 genes that were significantly differentially expressed (5% FDR) in all MCMC traces and sampled 100 of these to visualize the posterior distributions of the switch strength and switch time parameters (Supplementary Text Figure 5). This demonstrates that despite only fitting pseudotimes using marker genes it is possible to infer interpretable gene parameters for the entire transcriptome. Furthermore, this procedure is more statistically correct as it only uses the data once - a small set of genes to fit the pseudotimes with the remaining genes used for differential expression. This is in contrast to the majority of pseudotime procedures that use the data twice - once for pseudotime inference, and once again for differential expression, which will break asymptotic guarantees of the statistical tests.

# 8 Description of whole-transcriptome algorithms

**Monocle 2** Monocle 2 [6] begins with an initial dimensionality reduction using methods such as PCA and constructs a spanning tree in the reduced space, linking the centroids of clusters found using $k$-means clustering. Cells are then shifted towards the nearest tree vertex, with the spanning tree subsequently updated. The map is then projected back into high dimensional gene expression space, and the process repeated over until the tree and cell positions converge. A "root" cell is then selected by the user, and the distance from the root to any cell along the tree gives the pseudotime.

**Diffusion Pseudotime (DPT)** DPT [7] computes a transition matrix that approximates the probability that one cell would transition into another under a diffusion model, with cells closer together in expression space more likely to transition and conversely for cells further apart. The dominant eigenvectors of this transition matrix then act as a low-noise embedding of the cells, and scale-free random walks across this embedding are taken as the pseudotimes of the cells from a suitably defined root cell.

**TSCAN** TSCAN [8] begins by averaging genes into clusters to mitigate the effects of dropout. TSCAN then performs PCA on this reduced data matrix and retains the top $k$ components by fitting a breakpoint model to the variance explained per principal component. Cells are then clustered using Gaussan Mixture modelling where the number of clusters is chosen such that the BIC is maximized. TSCAN then constructs a minimum spanning tree on these clusters and cells projected onto this tree, with the distance from the root cell defining the pseudotime similarly to DPT.

# 9 Simulating data

Our method for generating synthetic pseudotemporally regulated single-cell RNA-seq data representing $\log_2(\text{TPM} + 1)$ given the above considerations can be found in algorithm 1.

**Algorithm 1** Generate pseudotemporally regulated scRNA-seq data

**Data:** $G$ genes, $N$ cells, $\phi = 3.5$, $\beta_0 = 1.76$, $\beta_1 = -1.16$
**Result:** A $N \times G$ matrix of gene expression $\mathbf{Y}$, where $[\mathbf{Y}]_{ng} = y_{ng}$
**for** $g \in 1 \dots G$ **do**
    Draw $k_g \sim$ Unif(5, 10)
    Draw $\eta_g \sim$ Unif$(2, 5)$
    Draw $t_g^{(0)} \sim$ Unif$(0, 1)$
    Set $k_g \leftarrow -k_g$ with probability $\frac{1}{2}$
    **for** $n \in 1 \dots N$ **do**
        Draw $t_n \sim$ Unif$(0, 1)$
        $\mu_{ng} \leftarrow \eta_g f(t_n, k_g, t_g^{(0)})$
        $\sigma_{ng}^2 \leftarrow \phi \mu_{ng}$
        Draw $x_{ng} \sim \mathcal{N}(\mu_{ng}, \sigma_{ng}^2)$
        **if** $x_{ng} < 0$ **then**
            $x_{ng} \leftarrow 0$
        **end if**
        $\theta_{ng} \leftarrow \text{logit}^{-1}(\beta_0 + \beta_1 \mu_{ng})$
        Set $x_{ng} \leftarrow 0$ with probability $\theta_{ng}$
    **end for**
**end for**

# 10 Incorporating prior information can improve pseudotime inference

A particular advantage of using Bayesian models with interpretable parameters is that we may express any prior knowledge about the gene behaviour as informative priors. For example, for each gene we model as switch-like there is the switch strength parameter $k$ that models how quickly a gene is upregulated if $k$ is positive or how quickly it is downregulated if it is negative. A researcher may have a firm prior belief that a gene will be up or downregulated along the trajectory and thus can place a prior $p(k)$ on the particular parameters. Using Bayes' rule, the posterior distribution of both the pseudotimes and gene-specific parameters is then calculated by combining this informative prior with the data likelihood. The crucial observation here is that the posterior distribution of the pseudotimes is affected by priors on the gene behaviour parameters, meaning incorporating prior information about gene behaviours may improve pseudotime inference. Such informative priors may be placed on any of the parameters that govern interpretable gene behaviour. For example, if a researcher

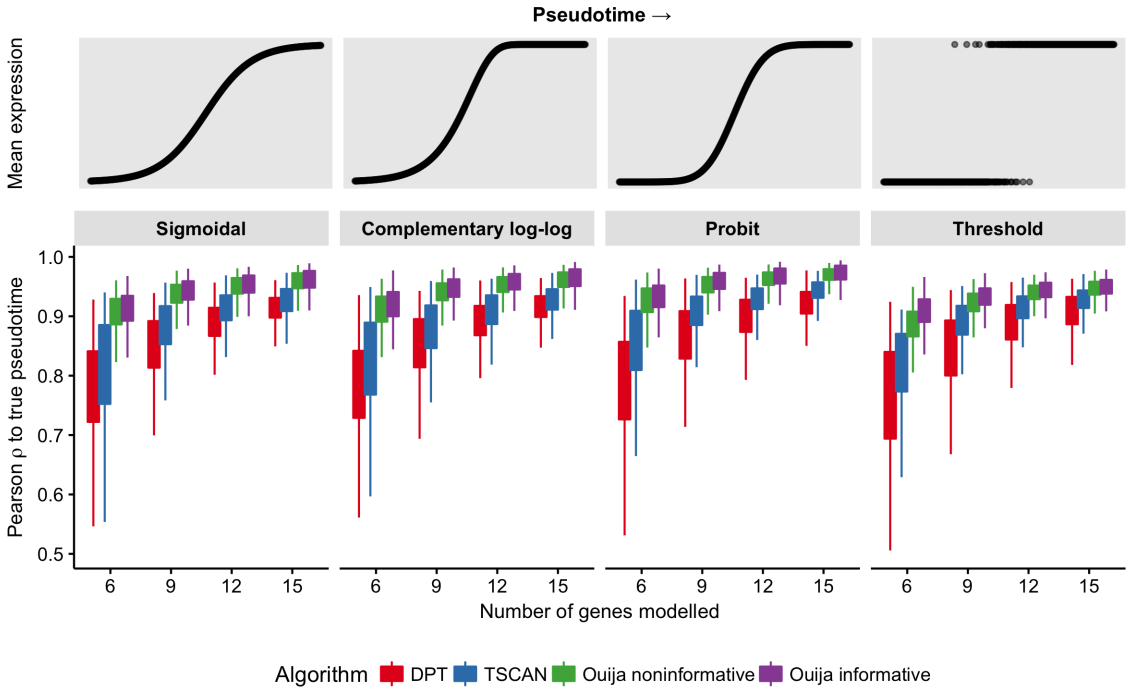


**Supplementary Text Figure 6: Incorporating prior information can improve pseudotime inference.** We sought to identify the benefits of incorporating prior information about the behaviour of genes to the accuracy of pseudotime inference. We simulated data according to four different mean functions (sigmoidal, complementary log-log, probit, and threshold) under identical noise model and reinferred using DPT, TSCAN, Ouija with noninformative priors, and Ouija with informative priors. The results show a marginal though significant gain in inference when incorporating prior knowledge.

expects a particular transient gene to peak early in the trajectory then they may encode this using a prior distribution on the peak time.

We sought to test the extent to which incorporating knowledge of gene behaviours through informative Bayesian priors aids pseudotime inference. To do so we performed extensive simulations of single-cell pseudotime under monotonic changes in expression and reinferred using Ouija with both noninformative and informative priors, as well as DPT and TSCAN. In order to emulate the fact that the data will not truly come from a sigmoidal link function, we simulated data from various link functions used in logistic regression including probit and complementary log-log (Supplementary Text Figure 6) along with a "threshold" model where the expression is on or off with a particular probability that changes along the trajectory (see supplementary text for full details).

The results can be seen in Supplementary Text Figure 6, with similar characteristics across the four mean functions considered. In all cases Ouija performs substatially better than DPT and TSCAN, but we note that this is likely due to the data generating model more closely matching the likelihood model of Ouija though could also be explained by the fact that DPT and TSCAN are not designed for small panels of genes. In each case the gain from incorporating prior information is statistically significant (Supplementary Table 3), but we note that the effect sizes are in practice quite small. Since to infer a consistent pseudotime, sufficient correlations must exist in the data, prior knowledge may only make a relatively minor contribution. However, researchers dealing with data with low biological signal to noise ratio may find it advantageous to incorporate such constraints to improve the quality of their inferences.

# 11 Benchmarking

## 11.1 Mean functions

We sought to simulated switch-like behaviour from four link functions commonly used in generalized linear regression, along with a modified version that leads to further misspecification.

### 11.1.1 Sigmoidal

The sigmoidal mean function corresponds to that used by Ouija. Given the pseudotime $t_n$ the mean is calculated via

$$\mu_{ng} = \frac{2\eta_g}{1 + \exp(-k_g(t_n - t_g^{(0)}))} \tag{5}$$

for which we draw $\eta_g \sim \text{Unif}(3, 4)$, $t_g^{(0)} \sim \text{Unif}(0.1, 0.9)$ and $k_g \sim \text{Unif}(5, 20)$ and negated with probability $\frac{1}{2}$. Given an assumed scale of $\log_2(\text{TPM} + 1)$ this selection of parame-

ters, combined with the noise model, provides a reasonable range of observed expression values.

#### 11.1.2 Complementary log-log

The complementary log-log (*cloglog*) acts as a link function in logistic regression, modelling the probability of success $\pi$ in terms of regressors $\mathbf{x}$ and coefficients $\boldsymbol{\beta}$ via $\log(-\log(1-\pi_n)) = \mathbf{x}_n^T \boldsymbol{\beta}$. Therefore, we use it to generate a mean via

$$\mu_{ng} = 2\eta_g \left(1 - \exp(-e^{k_g(t_n - t_g^{(0)})})\right) \tag{6}$$

for which we draw the parameters identically to the sigmoidal case.

#### 11.1.3 Probit

The probit link models the probability of success as $\pi_n = \Phi(\mathbf{x}_n^T \boldsymbol{\beta})$, where $\Phi$ is the cumulative distribution function of the standard normal distribution. Thus we use

$$\mu_{ng} = 2\eta_g \Phi \left(k_g(t_n - t_g^{(0)})\right). \tag{7}$$

$\eta$ and $t_g^{(0)}$ are drawn as before, while $k_g \sim \text{Unif}(10, 50)$ to take into account the differing curvature compared to sigmoid and cloglog (and negated with probability $\frac{1}{2}$ as before).

#### 11.1.4 Threshold

We sought to create a further switch-like mean function that would be maximally misspecified with respect to Ouija. This follows a two part process and requires a sign variable $|k_g|$ that describes whether the gene is up or down regulated along pseudotime. Firstly, a probit variable is generated by drawing $m_g \sim |k_g| \times \text{Unif}(1, 2)$ and $c_g = -m_g t_g^{(0)}$. Then draw $\mu_{ng}^* \sim \mathcal{N}(m_g t_n + c_g, 0.1)$. The mean function is given by

$$\mu_{ng} = \begin{cases} 2\eta_g & \text{if } \mu_{ng}^* > 0 \\ 0 & \text{otherwise} \end{cases} \tag{8}$$

In this situation we draw $\eta_g$ and $t_g^{(0)}$ as before and $|k_g|$ is positive or negative with equal probability.

## 11.2 Pseudotime inference

For each mean function we re-inferred the pseudotimes with PCA (first principal component), diffusion pseudotime, and Ouija in two configurations - noninformative and informative. For the noninformative case, default settings are used which consists of $k \sim \mathcal{N}(0,1)$ and $t^{(0)} \sim \mathcal{N}(0.5,1)$. For the informative case, the prior mean on $t_g^{(0)}$ was set to its true value and the prior standard deviation $0.1$.

For the informative case the prior on $k$ varied slightly depending on the simulation condition, but in each case the standard deviation was reduced to $0.1$. For the sigmoidal and cloglog mean function regimes, the prior means were set to the true values of the generated data. For the probit and threshold datasets, the prior was set to $50$ multiplied by the sign of the true $k$. In otherwords, we declare the direction of regulation, and that strong switch-like behaviour is exhibited, but nothing more.

We included a further two settings for Ouija on the sigmoidal dataset only. In the "switch midpoint" setting, the prior mean on $t^{(0)}$ is set to 0.5, while in "switch uncertainty" the prior mean on $t^{(0)}$ is set to the true value plus a $\mathcal{N}(0,0.1)$ random variable. In both cases, all other parameters are the same as the Ouija informative setting.

This data was simulated for $G = 6, 9, 12, 15$ "marker" genes with 500 replications per gene set and mean function.

# 12 Markers used

See supplementary table 4.

| **Dataset** | **Markers** |
|---|---|
| Trapnell et al. | *CDK1, ID1, MYOG, MEF2C, MYH3* |
| Zhou et al. | *Nrp1, Hey1, Efnb2, Ephb4, Nrp2, Nr2f2* |
| Shin et al. | *Sox11, Eomes, Stmn1, Apoe, Aldoc, Gfap* |
| Chu et al. | *POU5F1, NANOG, SOX2, EOMES, CER1, GATA4, DKK4, MYCT1, PRDM1, CDX1, MSX2* |
| Dulken et al. | *Id3, Clu, Rpl32, Egfr, Cdk4, Cdk1, Dlx2, Dcx* |
| Li et al. | *Mef2c, Foxa2, Col10a1, Comp, Dlx3, Id3, Pik3r2, Spry2, Sox9, Insig1, Scd1, Scd2* |

**Supplementary Table 4:** Markers used in the 6 datasets studies.

# References

[1] Junxiang Li, Haofei Luo, Rui Wang, Jidong Lang, Siyu Zhu, Zhenming Zhang, Jianhuo Fang, Keke Qu, Yuting Lin, Haizhou Long, et al. Systematic reconstruction of

molecular cascades regulating gp development using single-cell rna-seq. *Cell reports*, 15(7):1467–1480, 2016.

[2] Greg Finak, Andrew McDavid, Masanao Yajima, Jingyuan Deng, Vivian Gersuk, Alex K Shalek, Chloe K Slichter, Hannah W Miller, M Juliana McElrath, Martin Prlic, et al. Mast: a flexible statistical framework for assessing transcriptional changes and characterizing heterogeneity in single-cell rna sequencing data. *Genome biology*, 16(1):278, 2015.

[3] Emma Pierson and Christopher Yau. Zifa: Dimensionality reduction for zero-inflated single-cell gene expression analysis. *Genome biology*, 16(1):241, 2015.

[4] Carl Edward Rasmussen. Gaussian processes in machine learning. pages 63–71, 2004.

[5] John E Reid and Lorenz Wernisch. Pseudotime estimation: deconfounding single cell time series. *Bioinformatics*, 32(19):2973–2980, 2016.

[6] Xiaojie Qiu, Qi Mao, Ying Tang, Li Wang, Raghav Chawla, Hannah A Pliner, and Cole Trapnell. Reversed graph embedding resolves complex single-cell trajectories. *Nature methods*, 14(10):979, 2017.

[7] Laleh Haghverdi, Maren Buettner, F Alexander Wolf, Florian Buettner, and Fabian J Theis. Diffusion pseudotime robustly reconstructs lineage branching. *Nature methods*, 13(10):845, 2016.

[8] Zhicheng Ji and Hongkai Ji. Tscan: Pseudo-time reconstruction and evaluation in single-cell rna-seq analysis. *Nucleic acids research*, 44(13):e117–e117, 2016.
